# Severe Legionella and Histoplasma Pneumonia Acquired From Spring Water

**DOI:** 10.7759/cureus.34765

**Published:** 2023-02-08

**Authors:** Natalie G Street, Sohiub N Assaf, Shawnt Tosonian, Elman Urbina Meneses, Megan D Edwards

**Affiliations:** 1 Internal Medicine, The University of Tennessee Graduate School of Medicine, Knoxville, USA; 2 Medicine, The University of Tennessee Graduate School of Medicine, Knoxville, USA; 3 Pulmonary Critical Care Medicine, The University of Tennessee Graduate School of Medicine, Knoxville, USA; 4 Infectious Diseases, The University of Tennessee Graduate School of Medicine, Knoxville, USA

**Keywords:** acute respiratory distress syndrome (ards), community-acquired pneumonia (cap), ards, continuous positive airway pressure (cpap), legionnaires disease

## Abstract

Legionnaires’ disease and pulmonary histoplasmosis are important causes of community-acquired pneumonia. Environmental reservoirs remain the primary source of infection and may persist since investigations are often reserved for large outbreaks. Our case highlights a source of both legionella and histoplasmosis not previously reported. It demonstrates the value of taking a thorough history while recognizing non-traditional sources of both infections.

## Introduction

Legionnaires’ disease (LD) and pulmonary histoplasmosis are important causes of community-acquired pneumonia (CAP). Both infections are acquired via inhalation from a myriad of reported contaminated environmental sources. *Legionella pneumophila* and other Legionella spp. primarily inhabit aquatic environments, including natural bodies of water such as lakes and streams, as well as artificial water reservoirs. These organisms have the ability to exist in various forms within water, including as planktonic cells, as members of biofilms, or as intracellular parasites within protozoan hosts such as free-living amoebae and ciliates. This intracellular lifestyle provides protection from environmental fluctuations such as changes in temperature, pH, and nutrient availability. Optimal growth conditions for these organisms within water include warm temperatures ranging from 25 to 42°C, lack of water movement, and the presence of sediment [1]. The precise infectious dose required for human infection is yet to be quantified but is believed to be relatively high, with estimates suggesting that it may require the presence of over 1000 organisms [2]. The contamination of water sources with concentrations of Legionella sufficient to cause human infection can occur when changes in water flow or pressure disrupt biofilms and release large numbers of bacteria into the surrounding water. Furthermore, the ability of the Legionella bacterium to grow intracellularly within free-living amoeba may also facilitate transmission from water or soil. The incubation period for LD ranges from 2 to 10 days, with a median of 4 to 6 days [3,4]. 

The fungal pathogen Histoplasma capsulatum thrives in soil contaminated with avian or chiropteran excrement, which is thought to enhance soil conditions and augment nitrogen levels, thereby promoting the organism's growth. Frequently encountered sources of Histoplasma capsulatum include aviary structures or agricultural facilities with substantial accumulations of avian excrement, dilapidated structures, avian roosting sites, subterranean cavities, and wooded areas [5]. Activities linked to Histoplasma capsulatum exposure include excavation, construction, demolition, renovation, lumber harvesting and procurement, cave exploration, and cleaning of surfaces encrusted with avian or chiropteran excrement [6-12]. Herein, we present a case of concurrent LD and pulmonary histoplasmosis ultimately linked to contaminated spring water used in a home continuous positive airway pressure (CPAP) device, highlighting the importance of source identification and proper patient education on device maintenance. 

## Case presentation

**Figure 1:**
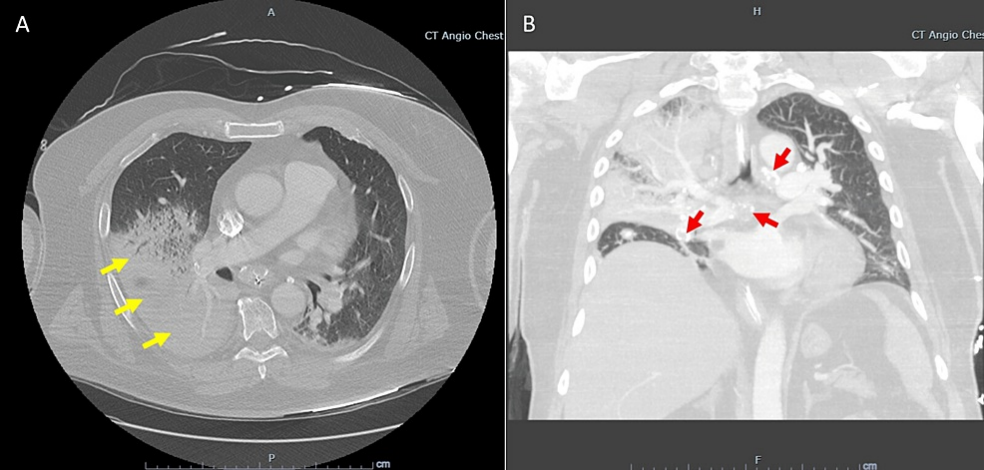
Computerized tomography of the thorax with contrast (A) Transverse plane computerized tomography with contrast demonstrating consolidation and airspace disease consistent with right greater than left pneumonia and atelectasis (yellow arrows). (B) Coronal plane computerized tomography demonstrating eggshell calcification evident throughout the mediastinal nodes (red arrows).

A 67-year-old male with obstructive sleep apnea was admitted to our intensive care unit (ICU) for acute respiratory distress syndrome (ARDS) requiring endotracheal intubation after one week of dyspnea associated with fevers, vomiting, and diarrhea. Vital signs on arrival were significant for a fever of 102.8°F, vasopressor-dependent hypotension, and refractory hypoxemia despite maximal ventilator support. Thoracic computerized tomography revealed bilateral multifocal consolidations (Figure 1), and the urine legionella antigen test was positive, confirming the diagnosis of LD. He was also diagnosed with possible pulmonary histoplasmosis after the serum histoplasma antigen result was positive. Treatment included salvage ARDS supportive measures and broad-spectrum antimicrobials, including levofloxacin and amphotericin B. His ICU course was complicated by a prolonged ventilator wean requiring tracheostomy. Upon further questioning, the patient’s family revealed that he used a nearby spring as his primary water source, including in his home CPAP device. He ultimately transitioned to a step-down unit with plans for outpatient pulmonary follow-up on discharge. 

## Discussion

Legionella pneumonia and pulmonary histoplasmosis both represent significant and potentially fatal causes of CAP. Environmental reservoirs remain the primary source of infection and may persist since investigations are often reserved for large outbreaks. Previous cases have described presentations of Legionella pneumonia associated with contaminated medical equipment; a few cases have described transmission from contaminated CPAP equipment colonized with legionella [13-15]. Recommendations regarding at home maintenance of CPAP equipment include using soap and water to disinfect all non-electronic surfaces including the reservoir tank which hold water for humidification of ventilated air. Patients with sleep apnea are at an increased risk of pneumonia, and the risk is further increased in patients treated with CPAP [16]. Our case highlights a source of both legionella and histoplasmosis not previously reported. It demonstrates the value of taking a thorough history while recognizing non-traditional sources of both infections. It also highlights the importance of emphasizing proper device maintenance. Increased awareness of risk factors for these infections enables earlier diagnosis and treatment and ideally improves outcomes. 

## Conclusions

Patient education regarding appropriate use and maintenance of CPAP machines is imperative to help reduce inadvertent transmission of pathogens through colonization of these devices. Recommendations currently include daily changes to water reservoirs with the use of distilled water strictly and avoiding outside contamination. 
